# Dog Ownership Enhances Symptomatic Responses to Air Pollution in Children with Asthma

**DOI:** 10.1289/ehp.8548

**Published:** 2006-08-29

**Authors:** Rob McConnell, Kiros Berhane, Jassy Molitor, Frank Gilliland, Nino Künzli, Peter S. Thorne, Duncan Thomas, W. James Gauderman, Edward Avol, Fred Lurmann, Edward Rappaport, Michael Jerrett, John M. Peters

**Affiliations:** 1 Department of Preventive Medicine, University of Southern California, Los Angeles, California, USA; 2 Department of Occupational and Environmental Health, College of Public Health, University of Iowa, Iowa City, Iowa, USA; 3 Sonoma Technology, Inc., Petaluma, California, USA

**Keywords:** air pollution, asthma, cats, child, dogs, endotoxin, epidemiology, indoor allergens, particulate matter

## Abstract

**Background:**

Experimental data suggest that asthma exacerbation by ambient air pollutants is enhanced by exposure to endotoxin and allergens; however, there is little supporting epidemiologic evidence.

**Methods:**

We evaluated whether the association of exposure to air pollution with annual prevalence of chronic cough, phlegm production, or bronchitis was modified by dog and cat ownership (indicators of allergen and endotoxin exposure). The study population consisted of 475 Southern California children with asthma from a longitudinal cohort of participants in the Children’s Health Study. We estimated average annual ambient exposure to nitrogen dioxide, ozone, particulate matter < 10, 2.5, and 10–2.5 μm in aerodynamic diameter (PM_10_, PM_2.5_, and PM_10–2.5_, respectively), elemental and organic carbon, and acid vapor from monitoring stations in each of the 12 study communities. Multivariate models were used to examine the effect of yearly variation of each pollutant. Effects were scaled to the variability that is common for each pollutant in representative communities in Southern California.

**Results:**

Among children owning a dog, there were strong associations between bronchitic symptoms and all pollutants examined. Odds ratios ranged from 1.30 per 4.2 μg/m^3^ for PM_10–2.5_ [95% confidence interval (CI), 0.91–1.87) to 1.91 per 1.2 μg/m^3^ for organic carbon (95% CI, 1.34–2.71). Effects were somewhat larger among children who owned both a cat and dog. There were no effects or small effects with wide CIs among children without a dog and among children who owned only a cat.

**Conclusion:**

Our results suggest that dog ownership, a source of residential exposure to endotoxin, may worsen the relationship between air pollution and respiratory symptoms in asthmatic children.

Evidence shows that ambient air pollution from combustion sources (Peden 2002), indoor allergens such as those from dogs, cats, and cockroaches (Institute of Mecidine 2000), and endotoxin (Michel 2003) all exacerbate asthma. In addition, increasing experimental evidence shows that oxidant air pollutants such as diesel exhaust particulate, nitrogen dioxide, and ozone enhance the effect of inhaled allergen on physiologic responses in the lungs of asthmatics and experimental animals (Diaz-Sanchez et al. 1999; Jenkins et al. 1999; Kehrl et al. 1999; Molfino et al. 1991). In recent studies, diesel exhaust particulate and O_3_ have also been shown to promote the endotoxin-induced inflammatory response in the lungs of animals (Johnston et al. 2002; Takano et al. 2002). However, there has been little epidemiologic evaluation of the effect on symptoms of asthma of co-exposure to ambient air pollution and endotoxin or indoor allergen exposure.

We hypothesized that exposure to indoor allergens and/or endotoxin would enhance the symptomatic response to exposure to ambient air pollution among children with asthma. In this study, we tested this hypothesis in the Southern California Children’s Health Study, a prospective study of air pollution and respiratory health. We previously reported that yearly variation in pollutant levels in the 12 Children’s Health Study communities was strongly associated with yearly variation in the prevalence of chronic cough, phlegm, and bronchitis among asthmatic children in this cohort (McConnell et al. 2003). Cough and bronchitis are nonspecific symptoms among asthmatic children that may represent an acute or chronic exacerbation, upper-airway cough syndrome due to rhinosinus conditions, or related conditions such as gastro-esophageal reflux disease (Pratter 2006). However, bronchitic symptoms are a sensitive end point for air pollution effects in population-based studies of children (Braun-Fahrlander et al. 1997). We showed that bronchitic symptoms were associated with air pollution only among children with asthma in this cohort (McConnell et al. 1999, 2003), results that are consistent with those of an earlier study (Dockery et al. 1989). We have now examined whether the effect of ambient air pollution on symptoms was larger among asthmatic participants who owned a dog or cat. Ownership of a dog or cat was used as a marker of indoor exposure to allergens and/or endotoxin, because pet ownership has been shown to be a strong predictor of the concentration of the respective allergen, commonly measured in house dust (Arbes et al. 2004). Although both cat and dog ownership have been associated with indoor endotoxin concentration in house dust, the association has generally been stronger and more consistent for dogs (Gehring et al. 2004; Heinrich et al. 2001; Thorne et al. 2002).

## Methods

The design of this longitudinal cohort study and the participants contributing to the current analysis have been described previously (McConnell et al. 2003). Briefly, participants in the Children’s Health Study, a population-based evaluation of air pollution and respiratory health, were recruited from schools in 12 communities in Southern California. A lifetime history of physician-diagnosed asthma was determined based on a questionnaire completed by a parent at study entry. A health questionnaire was administered yearly to children in classrooms. Our present study population included all 475 children with asthma among 3,227 participants in the cohort who completed two or more questionnaires between 1996 and 1999. The primary outcome of interest was the period prevalence of bronchitic symptoms, defined as having any one of the following: *a*) a cough first thing in the morning or *b*) at other times of day that lasted for as much as 3 months in a row during the previous 12 months; *c*) other than with colds, a child who usually seems congested in the chest or brings up phlegm; or *d*) a report of bronchitis during the previous 12 months.

Also reported yearly was information on the presence of secondhand tobacco smoke in the home (SHS) and personal smoking by the child. Additional information reported by parents on the questionnaire completed at study entry included ownership of a dog or cat, date of birth, sex, and race/ethnicity. We evaluated other characteristics that potentially could confound the interaction of pets and air pollution, including history of asthma in either parent, family socioeconomic status (SES), housing conditions, and outdoor activity. Families were considered to be of low SES if family income was < $15,000 (or, if income was not reported, if the responding parent had less than a 12th grade education). High SES was defined by family income of ≥ $100,000 (or, if income was not reported, by postgraduate training). Remaining families were classified as middle SES. Housing conditions included a history of mildew or mold or of water damage or flooding in the home while the child lived there, or of cockroaches in the home in the previous 12 months. Time reported spent outdoors was dichotomized for each cohort (1993 and 1996) into those children playing more than the median time outdoors and those playing less. (Time spent outdoors might increase the exposure to ambient air pollution and result in asthma exacerbation in more polluted environments.)

Air pollution monitoring stations were established in each of the 12 study communities. For each year of follow-up, measurements were made for each pollutant, as previously described (Gauderman et al. 2000; Peters et al. 1999). Each station monitored hourly levels of O_3_, particulate matter < 10 μm aerodynamic diameter (PM_10_), and NO_2_. PM < 2.5 μm aerodynamic diameter (PM_2.5_) and acid vapor were measured using 2-week integrated samplers. Elemental and organic carbon (EC and OC) were collected in 2-week integrated samples and subsequently analyzed by the National Institute for Occupational Safety and Health method (NIOSH 1996, 1999; Salmon et al. 2000). Annual averages were computed of the 24-hr PM_10_ and NO_2_, and of the 1000- to 1800-hr averages of O_3_. This O_3_ metric was selected because O_3_ has a marked diurnal pattern, with highest concentrations occurring during mid-day and afternoon periods, when children were likely to be outside and therefore more exposed. Annual averages also were computed from 2-week averages of PM_2.5_, of coarse PM_10–2.5_ (PM_10_ minus PM_2.5_), of inorganic hydrochloric plus nitric acid vapor, of organic acetic plus formic acid vapor, and of EC and OC. Four-year mean levels (1996–1999) in each community were computed for each pollutant metric. The yearly deviations from the 4-year mean were computed each year for each community.

The study was approved by the institutional review board at the University of Southern California, and informed consent was obtained from participants.

## Data Analysis

We examined the distributions of demographic and other characteristics by cat and dog ownership at study entry using descriptive statistics and tests for overall associations. The distributions of yearly temporal variation within communities in pollutants also were examined.

We used a multilevel modeling strategy that we have described previously (Berhane et al. 2004). We examined the effect of the yearly variability in air pollution levels on bronchitic symptoms, and we examined effect modification by dog and cat ownership and other covariates. To describe briefly a two-level logistic model used in this paper, let *c, i, j* denote the community, subject, and year of visit, respectively. In the first level, we examined the association between bronchitic symptoms and the deviation of yearly average air pollution from the 4-year average for each community, *X_cj_* − 
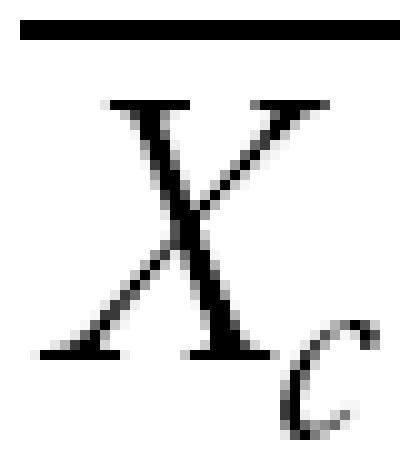
, where 
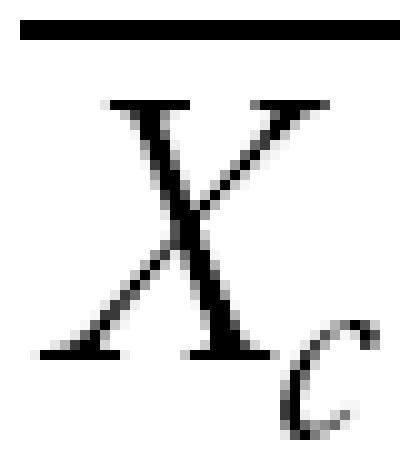
 represents the 4-year average level of air pollution for each community. This analysis included adjustments for time-dependent covariates *z_cij_* and estimated pollutant effects specific to pet owners and nonowners. The model thus has the following form (using dog ownership as an example):


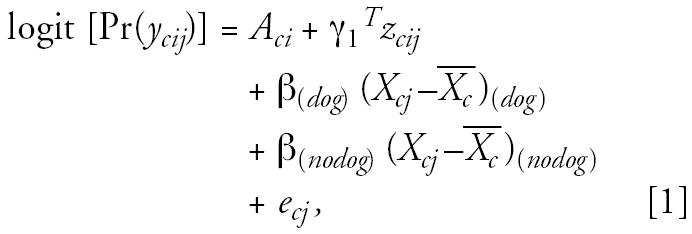


where


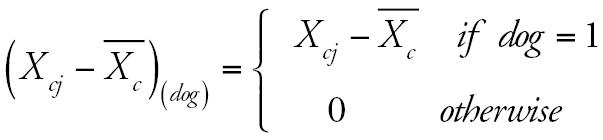


and


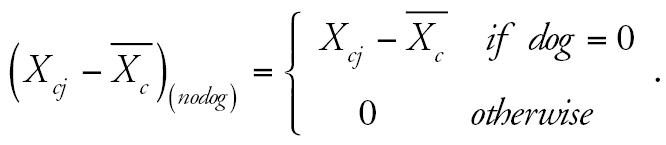


The interaction of *X_cj_* − 
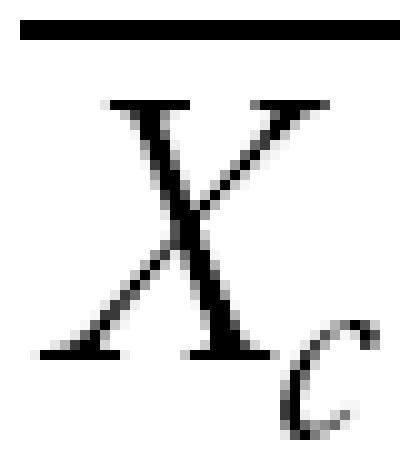
 with cat ownership and with potential confounders was also tested in an equivalent model. This model included subject-specific intercepts *A_ci_*, representing subject-specific adjusted log prevalence rates, to be used in the second-level models. Time-dependent covariates included age (centered at 12 years), children’s personal smoking history, and SHS exposure in the home.

In the second-level model, we used a linear regression model to adjust for effects of fixed subject-specific covariates *z_ci_*. This model has the following form:


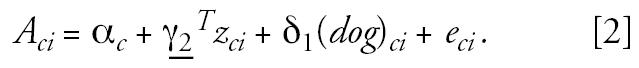


The covariates *z_ci_* included sex and race/ethnicity. This model included separate intercepts for each of the twelve communities to account for any community-level confounders.

These two regression models were combined to yield a more efficient logistic mixed-effects model of the form:


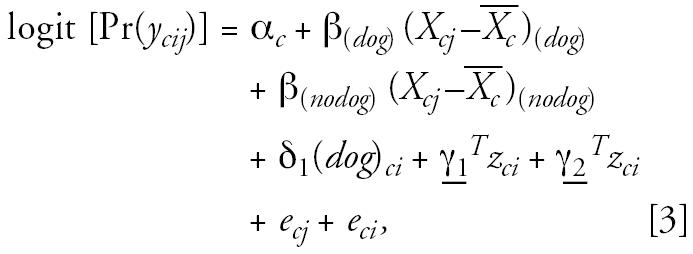


where *e_ci_* and *e_cj_* are random effects for subject and year, assumed to be randomly distributed with zero means and variances σ*_ci_*^2^ and σ*_cj_*^2^, respectively. The random effect for year, *e_cj_*, was included to account for effects of any temporal trends in pollution and symptoms. The parameters of primary interest were β(*dog*) and β_(_*_nodog_*_)_. These represent the dog stratum-specific effect on bronchitic symptoms of the yearly variation in air pollutants within communities. Children did not contribute to the analysis in years for which they were not available to complete the questionnaire. In all models, missing data were assumed to be missing completely at random (Diggle et al. 1994). We also examined stratum-specific effects of air pollution for homes with a dog only, with a cat only, with both pets, and with neither pet.

Because we were interested in how a pet in the home modified the effect of air pollution, we evaluated whether other exposures that might also interact with air pollution accounted for the effects of a dog or cat in the home. Thus, we assessed confounding of the interaction of dog (or cat) ownership with each pollutant by examining the change in the coefficient of this interaction after adjusting for potential confounding by other interactions (such as sex or parental history of asthma, for example) with air pollution. These interaction confounders were entered into the main model one variable at a time. Changes > 10% in the coefficient of interaction for pet ownership with the pollutant were considered to be evidence that effect modification by pets could be explained partially by another variable modifying the effect of air pollution.

Finally, we previously evaluated the main effects of between-community 4-year average pollutant levels on bronchitic symptoms in these children (McConnell et al. 2003). Here we therefore evaluated whether there was an interaction of pet ownership with average between-community pollutant levels, and we examined whether adjusting for these between-community effects of air pollution affected the within-community temporal variability, which is the primary focus of this analysis. To do this, we modified the second-level model to include community and pet ownership (*p*) specific intercepts (*A_cp_*) as follows:


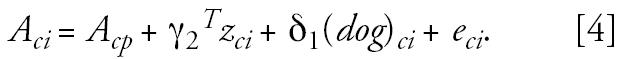


We then introduced a third-level model of the form


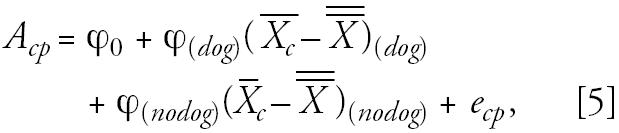


where ϕ_(_*_dog_*_)_ and ϕ_(_*_nodog_*_)_ denote the between-community effects of ambient air pollution (centered by the overall mean pollution levels) by individual pet (e.g., dog) ownership status.

All analyses were conducted using the SAS software version 8.2 (SAS Institute Inc. 1999). The GLIMMIX macro in SAS was used in fitting the logistic mixed-effects regression models. Statistical significance was assessed using a two-sided test at 5% level of significance.

## Results

Among study participants, 184 (39%) had bronchitic symptoms during the first year they contributed to the analysis. Dogs were present in 292 (62%) of homes and cats in 202 (43%) at study entry. Dogs were more common in the homes of boys and less common in the homes of children of low SES (Table 1). Both pets were more common in homes of non-Hispanic white children and in homes in which the parent reported mildew in the previous year. Few children smoked (*n* = 11). Children with dogs were similar in age (mean ± SD, 12.6 ± 1.95 years) to children without dogs (12.5 ± 1.73 years), as was the distribution of children with (12.6 ± 1.85 years) and without cats (12.6 ± 1.89 years).

Some communities had relatively little yearly variability in pollution (Table 2). In the community with the least yearly change in NO_2_, for example, between the least and most polluted year there was a difference of only 1.1 ppb. However, one community varied by almost 13 ppb in annual mean NO_2_ concentration between the least and most polluted year. In general, the most variable communities for each pollutant were also those with higher absolute pollutant levels (data not shown).

The association of yearly variability in each pollutant with bronchitic symptoms was consistently larger in children with dogs than without dogs (Table 3). A community with the median range in yearly variability for each pollutant (from Table 2) was selected to scale the effect estimates shown in Table 3 to represent variability that might be common for that pollutant in communities in Southern California. Among children who owned a dog, an increase in the period prevalence of bronchitis was associated with this variability for all pollutants. The smallest increase was observed for PM_10–2.5_ (30% per 4.2 μg/m^3^) and the largest for OC (91% per 1.2 μg/m^3^), followed by EC (74% per 0.29 μg/m^3^). These associations were significantly larger among children without dogs for PM_10_, PM_10–2.5_, EC, and both inorganic and organic acid. There was no consistent evidence for an effect of pollution among children without a dog. Among children with a cat, there were also generally larger effects of air pollution than among those without cats. However, the differences by strata of cat ownership were statistically significant only for organic acid.

We examined the possibility that the modifying effect of dog ownership observed in Table 3 was explained by the interaction of air pollution with some other potentially confounding exposure. The pattern of interaction effect estimates were, however, similar in models that also adjusted for the interaction of each pollutant with a cat or with sex, SES, mildew, water damage or cockroaches in the home, or time spent outside (results not shown). The dog × pollutant interaction effect estimates were generally stronger after adjusting for pollutant interactions with parental history of asthma and with ethnicity, and the interaction of dog with PM_2.5_, which was not significant in models not adjusted for these additional covariates, became significant. (Ethnicity was dichotomized into non-Hispanic whites and all others, because small numbers of children were from other groups.)

We also examined how the effect of air pollution was modified by dog ownership alone, cat ownership alone, or ownership of both cat and dog. (Table 4). The largest effects of air pollution were observed among children with both a cat and dog, and the largest effects in this group were observed for EC (odds ratio = 2.50) and OC (odds ratio = 2.22), although there were consistent positive effects among children with only a dog. There were no effects or small effects with wide confidence intervals for all pollutants among children without a dog and among children who owned only a cat.

Finally, we evaluated whether dog ownership modified the effect of 4-year average pollutant levels between communities. After adjusting for the interaction of dog with the temporal (within-community) variability in each pollutant, there was no modification of the effect of between-community pollutants on bronchitic symptoms (results not tabulated). However, neither the strength nor the pattern of within-community effect modification was changed by adjusting for between-community effects.

## Discussion

In this study, dog ownership modified the effect of exposure to air pollution in children with asthma. Yearly variation in multiple pollutants within the study communities was associated with the prevalence of bronchitis among children with a dog in the home. Effects of air pollution were greatest among children with both a dog and a cat, and effects were generally not observed among children with a cat alone. The effect of air pollution in homes with dogs was not explained by questionnaire-reported mildew, flooding or water damage, markers for damp housing and exposure to mold, and possibly house dust mite allergen (Gereda et al. 2001), or by other likely confounders. Therefore, some exposure associated with dogs may be important in augmenting the effect of air pollution.

The effect of dog might be explained by dog allergen or indirectly by an exposure such as endotoxin that is associated with dog ownership. Endotoxin is a component of the cell wall of gram-negative bacteria, and it has been suggested that early-life exposure to endotoxin may protect children from developing allergy and asthma by altering T-cell regulation (Eder and von Mutius 2004). However, inhaled endotoxin produces a marked inflammatory response in the fluid lavaged from the lungs (Jagielo et al. 1996), and in asthmatic subjects endotoxin exposure may cause bronchoconstriction (Michel 2003). In individuals who already have asthma, endotoxin may also contribute to airway remodeling and fixed obstruction in the airways (Reed and Milton 2001). Recently, endotoxin has been shown to enhance the inflammatory effect of inhaled highway aerosols (Elder et al. 2004), diesel exhaust particulate (Takano et al. 2002), and of ultrafine carbon particles and O_3_ in animals (Elder et al. 2000; Johnston et al. 2002; Wagner et al. 2003). This synergistic effect may occur because endotoxin promotes formation of reactive oxygen species and other free radicals after exposure to inhaled pollutants (Arimoto et al. 2005; Elder et al. 2000). In addition, the inflammatory response to endotoxin and to oxidant air pollutants may share common biologic pathways (Peden 2002; Wagner et al. 2003).

Dog ownership has been shown to be among the strongest predictors of increased endotoxin levels in house dust in many different cities with varying climate and levels of development (El Sharif et al. 2004; Gehring et al. 2004; Gereda et al. 2001; Heinrich et al. 2001; Park et al. 2001; Thorne et al. 2002; Waser et al. 2004, 2005; Wickens et al. 2003), although dog ownership has not universally been associated with higher endotoxin levels (Lau et al. 2005). Cats may also contribute to endotoxin levels in homes, but the association is not as consistent as for dogs (Bischof et al. 2002; El Sharif et al. 2004; Gereda et al. 2001; Park et al. 2001; Wickens et al. 2003), and dogs have generally been stronger predictors where homes with both dogs and cats have been studied (Gehring et al. 2004; Heinrich et al. 2001; Thorne et al. 2002). In one of the few studies that examined airborne endotoxin, homes with dogs had higher levels than homes with cats, even though there were not higher levels of endotoxin in settled house dust in homes with dogs (Platts-Mills et al. 2005). Cats are generally smaller animals, and dogs that walk or roll in endotoxin-laden soil may track more into the home. It has also been suggested that dogs are more likely to disturb house dust, which increases the levels of airborne endotoxin (Platts-Mills et al. 2005). There is limited evidence that higher endotoxin levels occur in homes with both pets than with either alone (Gereda et al. 2001). In our study, although the assessment of effects of air pollution in subgroups with different combinations of cats and dogs was limited by smaller numbers of children in these subgroups, the stronger effects observed in homes with both pets in Table 4 is consistent with an interaction of air pollution with endotoxin.

An alternative explanation for the larger effect of air pollution among children with a dog in the home is that allergen exposure was responsible for modifying the effect of air pollution. Asthmatic subjects allergic to house dust mite who were exposed experimentally to NO_2_ had larger decrements in forced expiratory volume in 1 sec (FEV_1_) if they were co-exposed to the allergen (Jenkins et al. 1999). Ragweed and ozone co-exposure among asthmatic subjects allergic to ragweed resulted in larger associated FEV_1_ decrements than did ragweed alone (Molfino et al. 1991). In another experiment, diesel exhaust particulate enhanced the ragweed-specific immunoglobulin E (IgE) in nasal lavage in response to allergen exposure (Diaz-Sanchez et al. 1997). Therefore, it is plausible that asthmatic subjects allergic to a pet might also have larger air pollution–associated exacerbation of symptoms if there were co-exposure to pet allergen in the home. The generally weaker interactions of cat ownership with air pollution and the absence of an effect of air pollution among children with only a cat in the home suggest that allergen exposure may be less likely to be responsible for the observed effects than endotoxin. Effect modification by allergen might be expected to occur among children allergic to the relevant pet. Allergy to dog dander assessed by IgE seropositivity is uncommon among asthmatic children in Southern California, and allergy to cat dander is common (Ferdman R, unpublished data).

Other interpretations of our results are possible, and additional information about allergy and exposure to pet allergen and endotoxin is needed to evaluate fully our hypothesis that endotoxin is responsible for the observed modification by dogs of the effect of air pollution on bronchitic symptoms. Atopic status to dog and cat allergen was not known for our study population, and only those allergic would be likely to respond to the respective allergen. The relationship between exposure, allergy, and asthma is complex and may vary by age of exposure, especially for ownership of cats, which has been shown to be protective for wheeze and asthma in some studies, perhaps depending on immunologic tolerance and age of exposure (Lau et al. 2005; Platts-Mills et al. 2001; Polk et al. 2004). In addition, cat allergen in clothing of children with cats has been reported to contaminate classrooms in diverse environments and to be carried home from the classroom by children without cats at home (Almqvist et al. 1999; Patchett et al. 1997). Cat allergen has also been shown to persist for prolonged periods in homes after the cat is gone (Wood et al. 1989). Although both cat and dog allergen are found in homes without pets, cat allergen is more commonly found in these homes at levels sufficient to provoke symptoms in sensitized asthmatics (Arbes et al. 2004). Therefore, children allergic to cats may have had significant exposure to allergen at home (or elsewhere in the community), regardless of cat ownership. This might result in smaller observed differences in the effect of air pollution associated with cat allergen exposure based on cat ownership. Indoor exposure levels to allergens (as well as endotoxin) may also be influenced by human and animal activity that may determine dust disturbance and the potential for respirable airborne exposure. There may also be other indoor interactions of interest that we could not evaluate. For example, one epidemiologic study has shown that the association of asthma severity with heavy house dust mite exposure was larger among participants with higher concentrations of endotoxin in house dust (Michel et al. 1996).

The effect of air pollution was remarkably consistent for a wide variety of pollutants. We previously reported positive main effects of within-community variation in air pollution for all pollutants, and these associations were significant for NO_2_, O_3_, PM_2.5_, and OC. Although only OC and NO_2_ demonstrated consistent independent effects in two-pollutant models (McConnell et al. 2003), the absence of a significant interaction of either of these pollutants with dog ownership in this new analysis may indicate that the effect was not attributable to a single pollutant. This is consistent with the known inflammatory effects of these pollutants and the central role of airway inflammation in asthma.

The effects of relatively small yearly variation in pollutants were remarkably large. The observed odds ratio of 1.60 for PM_10_ among children with dogs (for example), occurred for a modest 6.1-μg/m^3^ variation between years in average yearly exposure (from Table 3). One possible explanation is that the measured air pollutants were not those responsible for the observed effects in these children, but that they were indicators of other pollutants that varied relatively more between years than the pollutants we measured. Ambient ultrafine particulate matter, for example, has been hypothesized to be responsible for cardiorespiratory effects associated with NO_2_ (Seaton and Dennekamp 2003). Ultrafine particle number is known to vary markedly in the Los Angeles Air Basin (Sardar et al. 2004; Zhu et al. 2002). It is also possible that some other unmeasured pollutant with yearly variability that is correlated with the measured pollutants was responsible for the observed effect. Other factors that vary with the weather and other conditions that cause yearly variability in pollution could also explain the effects of air pollution. Exposures that deserve consideration include fungal spores and bioaerosols like pollen or pollen fragments, which may vary across years depending on rainfall (Delfino et al. 1996).

## Conclusion

Our results indicate that an increase in annual average ambient air pollution results in an increase in symptoms of bronchitis among asthmatic children, and that this effect occurs primarily among children with a dog in the home. An interaction between endotoxin and oxidant air pollutants is a plausible explanation. However, further research is needed to explore alternative hypotheses that could clarify the etiologic relationships and public health implications.

## Figures and Tables

**Table 1:** Distribution of demographic and other baseline characteristics by pet ownership [no. (%)].

Characteristic	Total^a^	Cat (*n* = 202)	Dog (*n* = 292)
Sex			
Girls	196	84 (43)	109 (56)*
Boys	279	118 (42)	183 (66)
Ethnicity			
Non-Hispanic white	279	150 (54)#	193 (69)#
Hispanic	124	39 (31)	68 (55)
Black	28	4 (14)	12 (43)
Asian	29	2 (6.9)	12 (41)
Others	15	7 (47)	7 (47)
Socioeconomic status			
Low	73	26 (36)	33 (45)#
Medium	330	138 (42)	219 (66)
High	68	37 (54)	37 (54)
Current SHS			
No	355	144 (41)	214 (60)
Yes	109	54 (50)	73 (67)
Personal smoking			
No	435	189 (43)	268 (62)
Yes	11	2 (18)	6 (55)
Parental asthma			
No	269	109 (41)	159 (59)
Yes	174	79 (45)	114 (66)
Mildew in home			
No	302	118 (39)*	176 (58)
Yes	164	82 (50)	111 (68)
Water damage			
No	378	155 (41)	228 (60)
Yes	92	45 (49)	60 (65)
Cockroaches			
No	420	181 (43)	259 (62)
Yes	40	15 (38)	25 (63)
Time spent outside			
Low	215	89 (41)	124 (58)
High	231	102 (44)	147 (64)

a Total in each category may not always sum to 475 for each characteristic, due to missing values. Distribution is for year child first contributed to the analyses.

* *p* < 0.05;

# *p* < 0.01

**Table 2:** Range of variability in the yearly deviation from the 4-year mean within each of the 12 communities.

Pollutant	Median^a^	Min–max
NO_2_ (ppb)	4.2	1.1–12.8
O_3_ (ppb)	4.3	1.7–13.2
PM_10_ (μg/m^3^)	6.1	2.3–14.7
PM_2.5_ (μg/m^3^)	3.4	0.89–8.7
PM_10–2.5_ (μg/m^3^)	4.2	1.3–9.7
Inorganic acid (ppb)	0.48	0.08–1.4
Organic acid (ppb)	0.77	0.30–2.1
EC (μg/m^3^)	0.29	0.08–0.74
OC (μg/m^3^)	1.2	0.50–2.9

a Median range of the deviation from the 4-year mean within each of the 12 communities; min and max are the ranges in the communities with the smallest and largest range of deviation from the community mean.

**Table 3:** Bronchitic symptoms and yearly variability in air pollution by dog and cat ownership [OR^a^ (95% CI)].

Pollutant	Dog (*n* = 292)	No dog (*n* = 183)	Interaction *p*-value^b^	Cat (*n* = 202)	No cat (*n* = 273)	Interaction *p*-value^b^
NO_2_	1.49 (1.14–1.95)	1.16 (0.84–1.60)	0.22	1.33 (0.95–1.86)	1.35 (1.04–1.76)	0.92
O_3_	1.41 (1.05–1.88)	1.09 (0.77–1.56)	0.25	1.41 (0.99–2.01)	1.19 (0.89–1.59)	0.44
PM_10_	1.60 (1.12–2.30)	0.89 (0.57–1.39)	0.02	1.47 (0.96–2.24)	1.20 (0.83–1.73)	0.41
PM_2.5_	1.56 (1.15–2.12)	1.03 (0.71–1.49)	0.06	1.30 (0.90–1.88)	1.35 (0.99–1.83)	0.87
PM_10–2.5_	1.30 (0.91–1.87)	0.76 (0.47–1.22)	0.05	1.37 (0.89–2.12)	0.92 (0.63–1.35)	0.13
EC	1.74 (1.16–2.61)	0.91 (0.58–1.42)	0.004	1.63 (1.01–2.62)	1.16 (0.79–1.70)	0.21
OC	1.91 (1.34–2.71)	1.07 (0.70–1.64)	0.16	1.66 (1.10–2.51)	1.43 (1.01–2.03)	0.56
Inorganic acid	1.40 (1.02–1.90)	0.74 (0.51–1.07)	0.01	1.36 (0.94–1.98)	0.95 (0.70–1.30)	0.10
Organic acid	1.30 (0.93–1.81)	0.91 (0.60–1.39)	0.02	1.55 (1.04–2.33)	0.92 (0.65–1.29)	0.03

a Odds ratio (OR) (95% confidence interval) per 4.2, 4.3, 0.48, 0.77 ppb for NO_2_, O_3_, and inorganic and organic acid, respectively; and per 6.1, 3.4, 4.2, 0.29, and 1.2 μg/m^3^ for PM_10_, PM_2.5_, PM_10_–PM_2.5_, EC, and OC, respectively (estimate scaled to range for median community; see “Results” and Table 2). All models were adjusted for age, SHS and personal smoking history, sex, and race.

b For air pollution effect with dog ownership or cat ownership, respectively.

**Table 4:** Bronchitic symptoms and yearly variability in air pollution by dog and cat ownership [OR^a^ (95% CI)].

Pollutant	Neither pet (*n* = 112)	Cat only (*n* = 71)	Dog only (*n* = 161)	Both pets (*n* = 131)
NO_2_	1.16 (0.79–1.71)	1.11 (0.61–2.01)	1.53 (1.08–2.16)	1.44 (0.96–2.15)
O_3_	1.09 (0.70–1.69)	1.07 (0.60–1.90)	1.26 (0.87–1.81)	1.63 (1.06–2.53)
PM_10_	0.91 (0.53–1.56)	0.84 (0.42–1.66)	1.41 (0.91–2.19)	1.89 (1.15–3.10)
PM_2.5_	1.11 (0.71–1.74)	0.85 (0.46–1.57)	1.53 (1.04–2.25)	1.58 (1.02–2.46)
PM_10–2.5_	0.72 (0.40–1.29)	0.84 (0.40–1.75)	1.06 (0.67–1.68)	1.69 (1.02–2.79)
EC	0.92 (0.54–1.57)	0.87 (0.43–1.75)	1.40 (0.87–2.25)	2.50 (1.37–4.58)
OC	1.13 (0.67–1.90)	0.97 (0.49–1.91)	1.70 (1.09–2.64)	2.22 (1.33–3.69)
Inorganic acid	0.72 (0.46–1.13)	0.73 (0.39–1.37)	1.15 (0.78–1.69)	1.79 (1.15–2.79)
Organic acid	0.75 (0.45–1.26)	1.24 (0.63–2.44)	1.04 (0.68–1.58)	1.72 (1.06–2.79)

a Odds ratio (OR) (95% confidence interval) per 4.2, 4.3, 0.48, 0.77 ppb for NO_2_, O_3_, and inorganic and organic acid, respectively; and per 6.1, 3.4, 4.2, 0.29, and 1.2 μg/m^3^ for PM_10_, PM_2.5_, PM_10_–PM_2.5_, EC, and OC, respectively (estimate scaled to range for median community; see “Results” and Table 2). All models were adjusted for age, SHS and personal smoking history, sex, and race.
